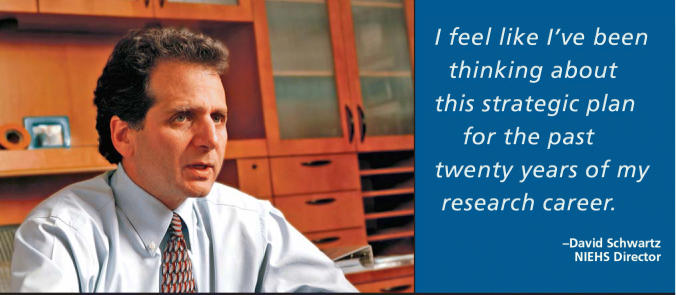# NIEHS Strategic Plan: New Frontiers in Environmental Sciences and Human Health

**Published:** 2006-05

**Authors:** Ernie Hood

The NIEHS has a rich history of scientific accomplishments and contributions to human health and well-being. As with any large-scale organization with far-reaching activities and widespread influence, however, it is advisable to take a step back periodically to critically examine mission, goals, objectives, strategies, and structure. With its recently completed strategic plan titled *New Frontiers in Environmental Sciences and Human Health: The 2006–2011 NIEHS Strategic Plan*, that is precisely what the institute has done. An intensive, inclusive process was designed to comprehensively and objectively reexamine, redirect, and in the end, reinvigorate the institute’s trajectory. Leaders hope the new plan will guide evaluation and decision making as the NIEHS strives to achieve its vision: “to use environmental sciences to understand human disease and improve human health.”

The plan lays forth an increased emphasis on leveraging scientific advances to benefit human health and longevity. “The plan will help us focus on the ultimate impact of our research in environmental health sciences,” says NIEHS director David Schwartz. “This direction is consistent with that of [former director] Ken Olden, builds on our strengths in environmental health sciences, and keeps us focused on human health and disease.”

In its final form, the plan is a blend of input from the many disparate stakeholders in the NIEHS research enterprise and Schwartz’s views about the role of the institute. Most observers have seen this mixture of leadership and outreach as appropriate and healthy. “It’s really important for the leader of an institute like the NIEHS to plant the flag, to lay out a vision of what he thinks is important, and [Schwartz has] done that in this strategic plan,” says Bernard Goldstein, an NIEHS National Advisory Environmental Health Sciences Council member who recently retired as dean of the University of Pittsburgh Graduate School of Public Health.

William Greenlee, president and CEO of the CIIT Centers for Health Research in Research Triangle Park, North Carolina, agrees. “I think where the differentiator between the NIEHS and other institutes within the NIH really comes [in the strategic plan] is the emphasis on dose–response and the emphasis on biomarkers that are relevant to interpreting exposure data. . . . I was wanting the NIEHS to make sure that it doesn’t appear to be another NIH disease institute, but that its environmental link is truly differentiated from the other NIH institutes. I think largely as I look through the plan, I see it there.”

The working group that formulated and guided the extensive strategic planning process was co-led by Sheila Newton, director of the NIEHS Office of Science Policy and Planning, and institute deputy director Samuel Wilson. Wilson says the institute expended considerable effort to gain substantial input from diverse members of the research community—experts from a broad range of disciplines, fields, and perspectives. He also cites “the very deliberate and systematic process we used in identifying those experts, and identifying a format within which we could obtain the information.” Adds Newton, “On the one hand, clearly the plan contains many of Dr. Schwartz’s ideas, but on the other hand, those ideas are not unique to him, and they reflect a lot of the thinking that’s been going on in our research community.”

## The Strategic Planning Process

The strategic planning process began with the formation of a working group consisting of more than 20 NIEHS staff members and local area investigators. The group, formed in June 2005, was charged with developing the procedures, format, and timetable for the overall process.

Following an announcement in the 21 June 2005 *Federal Register*, a six-question survey was posted on the NIEHS website, with a public comment period lasting until 5 August 2005. The survey generated more than 400 responses from academic and government scientists, advocacy groups, and individual citizens. After processing that input, and consulting with the NIEHS council, the group planned the next major event in the process—the Strategic Planning Forum, which was held 17–18 October 2005 in Chapel Hill, North Carolina.

More than 90 invited fundamental and applied scientists and public interest group members attended the event, which was cochaired by Frederica Perera, a professor of environmental health sciences and director of the Columbia Center for Children’s Environmental Health at Columbia University, and Gerald Wogan, an emeritus professor of toxicology and chemistry at the Massachusetts Institute of Technology. Attendees were assigned to rotating breakout discussion groups, with each group asked to discuss one of six core topics related to future NIEHS priorities. Conclusions from each group were then presented to the entire assembly in periodic plenary sessions.

The deep and lively discussions at the forum generated an enormous amount of input. “The meeting showed that all of the people who attended were engaged in the process,” says Perera, “and really worked hard in the different sessions to help shape the strategic plan.” After the forum, the input was analyzed by NIEHS staff and advisors, and a formal “proceedings” document was generated.

Newton describes the input gathered from the web survey as remarkably consistent, with many important themes articulated, including fostering training opportunities for future environmental health researchers at all educational levels and the critical need for validated biomarkers. “Many of those themes were reaffirmed, for the most part, by an entirely different group of people at the forum,” she says. “In its own way, that’s remarkable, and gives us a lot of confidence that we have a document that we can trust as we move forward.”

Following additional discussions with members of the NIEHS Public Interest Liaison Group (which includes representatives of disease groups, at-risk groups, and environmental groups who meet periodically with NIEHS staff), a draft of the strategic plan was posted on the NIEHS website in December for public comment through 24 January 2006. Revisions were made to the document reflecting the comments received, and the updated plan was presented to the NIEHS National Advisory Environmental Health Sciences Council at its February 2006 meeting (this group advises the NIEHS on research issues and programmatic content). The final document, incorporating the comments and discussion generated at the council meeting, was completed in March.

## A New Outlook

Wilson says the plan will have a large impact on the way the institute does business. “We are going through a period now of careful analysis of the existing programs and the potential for new programs,” he says, “and the guidance that we can obtain from this strategic plan will be critically important in this process of planning and priority setting.”

The new outlook described in the strategic plan involves an increased emphasis and sharpened focus on understanding how environmental exposures affect human biology, and on applying that knowledge to reduce morbidity and mortality. As stated in the plan, “Experience tells us that virtually all human diseases can be caused, modified, or altered by environmental agents. . . . The NIEHS is in a unique position to focus on the interplay between environmental exposures, vulnerable populations, human biology, genetics, and the common diseases that limit our longevity and quality of life.” If the NIEHS is to take advantage of this position, however, it must meet three challenges, as identified in the strategic plan.

The first challenge involves programmatic scope—the need to focus the research portfolio on those diseases and exposures that will optimize the future utility of the research for the greatest impact on human health. This prioritization will be pursued while the institute continues to fund innovative research efforts aimed at identifying new diseases with an environmental component as well as more classical research looking at the potential health implications of emerging environmental exposures. The second challenge involves the concept of integrative science, which the plan states will require a change in the institute’s approach to basic research, “moving from our traditional science base of single investigators with a clear hypothesis to integrated research teams addressing the complex hypotheses associated with the interplay of environmental factors with many other factors (e.g., genetics, lifestyle, age, sex) on disease incidence and prognosis.” The third challenge involves the public health impact of institute research findings, at both the individual and societal level. As the plan puts it, “How will we develop the scientific knowledge that empowers people to improve their environmental choices, [and] allows society to make appropriate public health decisions and results in our living healthier lives?”

None of these are new challenges for the NIEHS. But in seeking to maximize the benefits of research investments in improving the nation’s health, the enhanced efforts described in the strategic plan are clearly directed at improving and accelerating the translation of new environmental health science knowledge to new therapeutic and preventive modalities. This more focused paradigm is embodied in seven broad goals, each supported by more specific objectives.

## Statement of Goals

The first of the seven goals is to “expand the role of clinical research in environmental health sciences.” Under that rather broad umbrella, the institute will seek to encourage clinical research that emphasizes the use of environmental exposures to understand and better characterize common, complex diseases; develop improved models for human disease using our knowledge of environmental exposures and human biology; and enhance the role of the clinical investigator in environmental health sciences, bringing in both physicians and PhDs.

The institute is already taking steps to implement the goal of expanding clinical research. As noted in the strategic plan, it has established the Outstanding New Environmental Scientist (ONES) award to fund first-time R01 recipients who are using environmental science to understand a human disease. Also, the institute plans to establish a Clinical Research Unit within its Division of Intramural Research.

Goals II (“use environmental toxicants to understand basic mechanisms in human biology”) and III (“build integrated environmental health research programs to address the cross-cutting problems in human biology and human disease”) elaborate on the plan’s overarching theme of the need for clinical research to more pointedly explore the relationship between environmental exposures and human disease, making full use of the new tools and technologies available, while encouraging the development of new ones. Some feel that the field is on the brink of a period of unprecedented and extraordinarily valuable discoveries. “My very strong view,” says Schwartz, “is that environmental health science is poised to make incredibly important contributions to understanding very basic biological mechanisms that will have profound effects on human health and disease.”

Goal III and its objectives encourage the promotion of integrative, interdisciplinary research models, with basic and applied investigators working together collaboratively on specific questions. This approach is seen as a way to increase the relevance, productivity, and impact of NIEHS research programs. “Ultimately, we want all of this research to lead toward something significant beyond a report in a journal,” says Newton. “And a lot of the questions that we have now, that we really need answers to, require cross-fertilization and better collaboration between groups from different disciplines.”

The strategic plan announced a concrete step in pursuit of Goal III—the development of a new program called Disease Investigation for Specialized Clinically Oriented Ventures in Environmental Research (DISCOVER). DISCOVER is designed to bring together basic, clinical, and population-based scientists to conduct integrative research programs on understanding the etiology and pathogenesis of human diseases influenced by environmental factors, using exposure to understand the interplay between genetic and environmental factors, and applying available state-of-the-art technologies and methods to improve human health.

Goal IV is “improve and expand community-linked research.” The NIEHS has taken a lead role both in investigating environmental influences on disease in minority and socioeconomically disadvantaged populations and in developing tools and strategies to reduce health disparities. The report states, “We will continue to support research, both domestically and globally, that can offer important insights into how to reduce exposures and disease incidence in these community settings. . . . The likelihood of exposure to environmental toxicants increases in most economically disadvantaged communities and is associated with an excess disease burden in these communities.”

Throughout the strategic planning process, the urgent need to develop new biomarkers of exposure, susceptibility, and effect, along with the technological advances in exposure assessment to allow their discovery, came through loud and clear. As expressed in Goal V of the plan, improvement in exposure assessment has to be one of the institute’s top priorities. Says Wilson, “The need for quantitative measures of exposure is paramount in the environmental health sciences, and has been for many years. And certainly as we move toward more gene–environment type research, the quantitative measure of environmental exposure is absolutely fundamental.”

The recently announced NIH Genes and Environment Initiative will be a first step toward achieving the goal of improved exposure assessment. The initiative constitutes a major federal investment in the development of innovative new technologies to measure environmental toxicants, dietary intake, and physical activity, and to determine an individual’s biological response to those influences. The environmental arm of the project will be spearheaded by the NIEHS.

Goals VI (“recruit and train the next generation of environmental health scientists”) and VII (“foster the development of partnerships between the NIEHS and other NIH institutes, national and international research agencies, academia, industry, and community organizations to improve human health”) reflect common themes heard at all stages of the strategic planning process. The pursuit of partnerships, particularly to improve access to diverse subject populations and data sets, is widely endorsed, although some observers note that it is important that the NIEHS maintain its distinctive identity as it reaches out to other agencies. Nsedu Obot Witherspoon, executive director of the Children’s Environmental Health Network in Washington, DC, summarizes this sentiment: “There’s a fine line between what the NIEHS specifically brings as its own novelty versus what the overall NIH does. We need to make sure that we consistently work in a check-and-balance type of system, to ensure that we don’t completely lose the unique entity that the NIEHS has [been] by leading environmental health research in the United States.”

## Kudos and Caveats

Observers contacted for their reactions to the strategic plan unanimously supported the overall goals and objectives outlined in the document. Several specifics also met with a warm reception. For instance, Greenlee was pleased to see the plan’s emphasis on exposure assessment. “It’s great to understand the biology,” he says, “but you have to be able to put it in a . . . context of how external perturbations or exposures translate quantitatively into dose–response changes, and [then] target tissues, and then of course integrate that quantitatively with knowledge of biology to understand how that leads to potential health outcomes.”

Witherspoon commended the plan’s inclusion of continuing and expanding community-linked research as an individual goal. The question, she says, is how can stakeholders be effective resources for the institute, to assist the institute in being the most effective resource for various communities across the country?

John Balbus, director of health programs at the advocacy organization Environmental Defense, says, “I’m pleased to see the strategic plan developing in a way that reflects the importance of community-based research programs and basic toxicology, yet provides a sharper focus on diseases with the greatest public health burdens.” While there are still details to be worked out, he adds, this plan provides a good framework for merging newer analytic tools with traditional ones in meeting the ultimate goal of the NIEHS in preventing disease due to environmental causes.

Deborah W. Brooks, president and CEO of The Michael J. Fox Foundation for Parkinson’s Research, endorses the interdisciplinary approach of the plan, but adds a cautionary note. “It’s not enough to [call them] interdisciplinary teams, and then just continue the work as usual,” she says. “To make interdisciplinary teams most powerful, you want a different kind of end point, which is a specific goal, a deliverable, an outcome. You want to empower those various experts at different points along a translational continuum to really think about how to problem-solve and get to an end point.”

Goldstein also wants to ensure that the historic strengths of the institute in public health and prevention are not diminished by the new directions outlined in the plan. “The NIEHS in the past has made its major impact on human health through its translation to public policy, not through its translation to the bedside,” he says. “I don’t disagree with putting emphasis on clinical disease, but the question is, will the emphasis remain on what has basically been the glory of the NIEHS—what it’s been able to do to prevent disease?”

## A New Chapter

With the strategic plan now in place, the process of implementing its far-ranging ideas begins, and the eyes of the environmental health sciences community will be on the NIEHS to assess how effective that implementation will be, and what its impact will be upon the many constituencies served by the institute. “Certainly, we’ll have to wait and see what happens once the new programs are put in place,” says Fernando Martinez, a professor of pediatrics at the University of Arizona in Tucson. “With that caveat, I think the general ideas that were discussed and the specific strategies that have been proposed will move very strongly and very appropriately toward this new approach, this crucial new orientation for the NIEHS.”

Although Schwartz says, “I feel like I’ve been thinking about this strategic plan for the past twenty years of my research career,” he emphasizes that the plan is not fixed in stone. The process of seeking and incorporating input and assessment will continue. Wilson calls this “the lifeblood of how we do business—gaining advice, understanding, and perspective from a very broad range of scientists and others involved with the institute.”

Schwartz stresses that “although the plan seems like a finalized process, it’s really just the beginning—the beginning of a lot of exciting work, a lot of exciting program development, and a lot of opportunity. We view this as a way of communicating very clearly as to what we think are our priorities for growth in May 2006, but we encourage our constituents to help us identify new priorities and new opportunities as they evolve.”

## Figures and Tables

**Figure:**
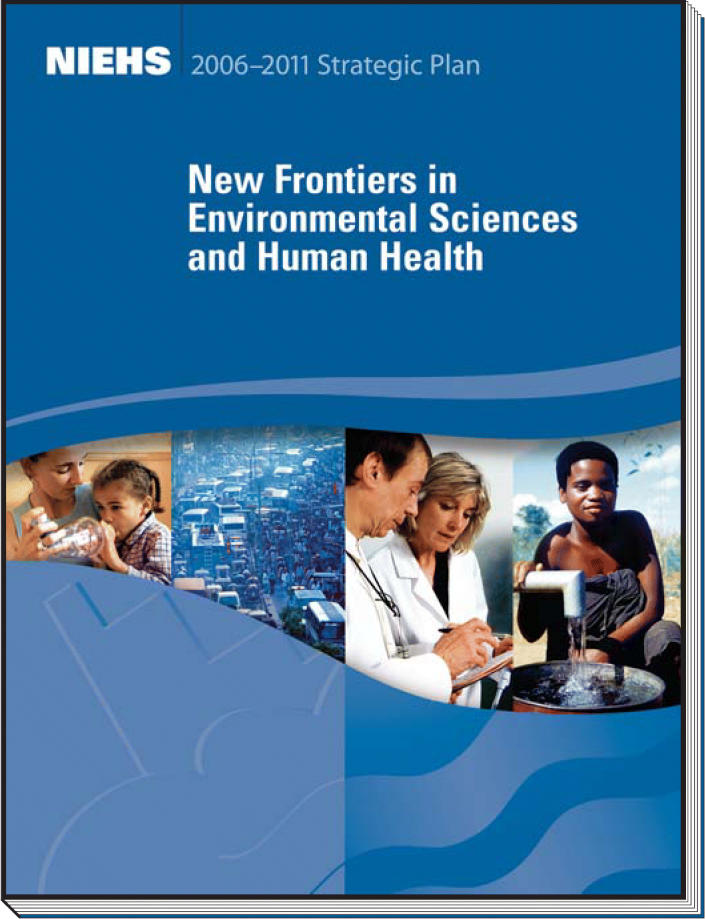


**Figure:**